# Justifying model complexity: Evaluating transfer learning against classical models for intraoperative nociception monitoring under anesthesia

**DOI:** 10.1371/journal.pone.0342688

**Published:** 2026-02-17

**Authors:** Chanseo Lee, Jaihyoung Lee, Kimon-Aristotelis Vogt, Muhammad Munshi

**Affiliations:** 1 Yale School of Medicine, New Haven, Connecticut, United States of America; 2 Sporo Health, Boston, Massachusetts, United States of America; 3 Paulson School of Engineering and Applied Sciences, Harvard University, Boston, Massachusetts, United States of America; Sreenidhi Institute of Science and Technology, INDIA

## Abstract

**Background:**

Accurate intraoperative detection of nociceptive events is essential for optimizing analgesic administration and improving postoperative outcomes. Although deep learning approaches promise improved modeling of complex physiologic dynamics, their added computational and operational complexity may not translate into clinically meaningful benefit, particularly in small, high-resolution perioperative datasets.

**Methods:**

We performed a head-to-head evaluation of classical supervised models (L1-regularized logistic regression and 50-, 200-tree Random Forests, with and without drug dosing features) against a Temporal Convolutional Network (TCN) transfer-learning framework for intraoperative nociception detection. Using 101 adult surgical cases with 30 physiologic and 18 drug dosing features sampled in 5-second windows, models were assessed under leave-one-surgery-out cross-validation using AUROC and AUPRC. We further examined probability calibration, multiple ensemble strategies, permutation importance features, and computational cost in terms of inference operations and memory footprint.

**Results:**

Drug-aware Random Forests of various trees (50 trees vs. 200 trees) achieved the highest discrimination (AUROC 0.716; AUPRC 0.399), outperforming the TCN transfer-learning model (AUROC 0.649; AUPRC 0.311). However, increasing personalization windows in the TCN yielded inconsistent and modest gains (p > 0.05). Isotonic calibration substantially improved probability calibration but did not affect discrimination. No ensemble method surpassed the standalone Random Forest; the gated network consistently assigned >84% weight to the classical model. Computational analysis revealed that while the TCN was more compact in total memory footprint, the smaller, 50-tree Random Forest inference required two orders of magnitude fewer operations, with faster training and lower operational complexity.

**Conclusions:**

In this clinically realistic benchmark, interpretable classical models operating on well-engineered features without personalization matched or exceeded the performance of a personalized deep learning approach while remaining computationally cheaper and simpler to deploy. These findings underscore the importance of rigorously justifying model complexity in perioperative machine learning and suggest that, for intraoperative nociception monitoring, classical approaches may offer a more favorable balance of accuracy, interpretability, and operational efficiency.

## Introduction

Accurate intraoperative detection of nociceptive events is critical for optimizing analgesic administration, maintaining hemodynamic stability, and improving postoperative outcomes. Inadequate nociception monitoring during general anesthesia has been associated with both acute complications and long-term sequelae, including poorly controlled postoperative pain and chronic pain syndromes [1,2]. Despite its clinical importance, nociception remains difficult to quantify intraoperatively due to the subjective nature of pain and the complex interaction between autonomic physiology, anesthetic depth, and pharmacologic interventions.

Recent advances in machine learning have motivated the development of algorithmic approaches to infer nociception from multimodal physiologic signals. Notably, proprietary systems such as the Nociception Level (NOL) monitor have demonstrated clinical benefit in select settings, including reductions in postoperative pain scores [3]. However, these systems rely on specialized hardware and closed-source pipelines, limiting transparency, reproducibility, and broader clinical adoption. In parallel, a substantial body of work has demonstrated that classical machine learning methods, such as Random Forests and logistic regressions, robustly discriminate pain-related states from engineered physiologic features such as electrodermal activity and cardiovascular indices, while maintaining interpretability and ease of deployment [4,5].

At the same time, deep learning architectures have gained increasing attention for nociception detection due to their ability to model complex temporal and multimodal relationships. Convolutional and recurrent neural networks integrating signals such as EEG, PPG, and ECG have reported improved discrimination over single-sensor approaches in some settings [6]. However, these gains often come at the cost of increased model complexity, greater computational burden, and heightened risk of overfitting, particularly in perioperative datasets that are small, imbalanced, and heterogeneous [7]. As a result, recent literature’s stance remains unclear whether the additional architectural sophistication of deep learning models translates into meaningful clinical benefit over well-engineered classical approaches [8].

Transfer learning has been proposed as a potential solution to this trade-off [9], enabling deep models to adapt rapidly to individual patients using limited additional data. In theory, patient-specific fine-tuning could improve personalization without requiring large datasets. However, few studies have rigorously evaluated whether such personalization meaningfully improves performance in realistic intraoperative settings, or whether its benefits justify the added complexity.

Critically, existing literature has largely evaluated classical and deep learning approaches in isolation, often under different validations or datasets. This fragmentation led to one fundamental question: when does increase model complexity provide value in perioperative machine learning? Moreover, prior work has rarely quantified computational considerations such as inference cost or memory footprint despite their central importance for real-time clinical deployment.

In this study, we address these gaps through a head-to-head benchmark of classical supervised models and a transfer-learning deep learning framework for intraoperative nociception monitoring. Using a publicly available, clinically annotated dataset and a Leave-One-Surgery-Out cross-validation design, we directly compare L1-regularized logistic regression, Random Forests, and a Temporal Convolutional Network (TCN) with patient-specific adaptation. Beyond predictive performance, we systematically examine probability calibration, employ multiple ensemble strategies, and analytically characterize computational cost in terms of inference operations and memory requirements.

This study is especially relevant in the context of large language models and AI pipelines gaining traction in medical tasks. The emergence of these computationally expensive models raise substantial concerns regarding transparency, ethical data risk, and resource allocation [10,11]. In fact, while emerging foundations like TabPFN show that transformer-based tabular models can excel in small-data regimes, their applicability to high-resolution time-series physiologic data remains both untested and computationally demanding without obvious benefit [12].

Our findings highlight that well-curated feature sets and interpretable classical models can match or exceed the performance of complex deep learning frameworks on nociception detection, while dramatically reducing computational burden and enhancing clinician trust. These findings are supported by several prior literature, although the subject remains hotspot for debate [7,8,11,13–16]. Our study underscores the importance of not just evaluating, but justifying model complexity as the medical community explores deep learning architectures and LLMs for healthcare problems [17].

## Methods

### Data source and pre-processing

The dataset was sourced from PhysioNet [18,19]. Subramanian et al. compiled a prospective archive of multi-sensor, continuous physiologic recordings (derived from ECG, EDA) and real-time drug dosing from 101 adult surgical cases, paired with manual annotations by anesthesiologists of 50,000 surgical nociceptive stimuli across ~18,500 minutes of surgery. 15 autonomic features and their respective estimated first derivatives for a total of 30 physiologic features, and 18 drug dosing chronology covariates (time since dose, cumulative dose) from nine anesthetic drug classes. Each feature is available for reader review in the original PhysioNet dataset.

The data was concatenated into a single-table with non-overlapping 5-second windows and then underwent quality assurance checks (e.g., zero imputation for missing values in drug dosing). Every numeric column was then standard‑scaled across the entire pooled cohort to zero mean and unit variance, ensuring equal weight during model fitting. For reproducibility, we created two input matrices: a 48‑column version that includes both physiologic and drug dosing features (drug-aware), and a 30‑column version that includes only physiologic (drug-naïve). The manual annotated nociceptive stimuli recordings were used as the ground truth for comparison.

Thus, drug-aware models trained from these two tables included 48 total input features, with 18 drug dosing features derived from nine drug classes, consisting of cumulative dose and time-since-last-dose for each agent. Drug-naïve models excluded all drugs and used only the 30 physiologic features. All covariates were standardized to zero mean and unit variance prior to model training.

### Model creation and performance evaluation

Each model’s creation and performance were completed using a Leave-One-Surgery-Out (LOSO) cross-validation strategy. In this approach, data from each surgery was held out in turn as the test set, while the models were trained and saved on the remaining surgeries. This process was repeated for all surgeries, ensuring that each subject contributed exactly once as a test case.

For each LOSO fold, the held-out surgery was further partitioned for transfer learning experiments. For transfer-learning models, the initial segment of the surgery was used for patient-specific adaptation (fine-tuning), while the remainder was reserved for evaluation. The adaptation window was varied to assess the impact of patient-specific data on model performance.

Model discrimination was quantified using the Area Under the Receiver Operating Characteristic Curve (AUROC) and the Area Under the Precision-Recall Curve (AUPRC). 95% confidence intervals for the median AUROC and AUPRC were calculated using non-parametric bootstrapping with 10,000 resamples. To assess the statistical significance of differences between models, pairwise comparisons of AUROC and AUPRC distributions were performed using non-parametric Wilcoxon signed-rank test.

### Producing benchmark supervised models

To establish a performance benchmark, we created baseline models based on Subramanian et al. [1] Four models were implemented: two logistic regression models with L1 regularization (LASSO), selected via the Akaike Information Criterion (AIC), with and without inclusion of pharmacologic features; and two random forest classifiers, each consisting of 200 decision trees with a maximum depth of 50, trained using 90% bootstrap resampling to mitigate overfitting. For ensemble experiments, an additional 50-tree random forest classifier with drug information was also trained with the same methodology.

### Transfer-learning models with adaptive windows

Each transfer-learning experiment was structured as a two-phase, leave-one-subject-out protocol. First, a global base model was initialized by pooling all 5-second windows from 100 of the 101 surgeries and training a lightweight Temporal Convolutional Network (TCN). This network applies a single 1-D convolution across the feature channels (48 channels when drug covariates are included, 30 otherwise), followed by batch-normalization, ReLU, global max-pooling, and a two-layer dense head. We optimized all parameters for up to twelve epochs (Adam, α = 1 × 10 ⁻ ³, batch = 128) with binary cross-entropy loss weighted for the 6% event prevalence, using early stopping (patience = 3) to avoid over-fitting. The resulting weights were checkpointed as the base model for that LOSO fold.

In the personalization phase, we loaded the base model, froze its convolutional and normalization layers, and fine-tuned only the dense head on the first K minutes of the held-out patient’s own data (where K ∈ {1, 2, 5, 10}, corresponding to 12, 24, 60, or 120 windows). Fine-tuning ran for three epochs (Adam, α = 1 × 10 ⁻ ⁴, batch = 128) to prevent forgetting and optimizing for small dataset size. The resulting models were saved for each patient in a LOSO-fashion.

### Calibration analysis

To assess the reliability of predicted probabilities from the transfer learning models, calibration analysis was performed across adaptation window lengths. For each window, the TL model was evaluated on the held-out portion of each surgery in the LOSO cross-validation framework. Predicted probabilities and true labels were aggregated for each adaptation window.

Three calibration approaches were compared: raw (uncalibrated), in which direct probabilities are output from the TL model; Platt scaling, in which a logistic regression model was fit to map the raw outputs to calibrated probabilities; isotonic regression, in which a non-parametric isotonic regression model was fit to the raw outputs.

Calibration performance was assessed using reliability curves, the Brier score, and the expected calibration error (ECE), computed with 10 quantile-based bins. For each method, calibration curves were plotted by comparing the mean predicted probability to the observed event frequency within each bin.

### Ensemble methods

To further enhance predictive performance, several ensemble strategies were evaluated by combining the outputs of the RF and TL models. First, a simple linear combination was implemented, where the final prediction was a weighted average of the RF and TL model outputs, with weights either fixed or optimized via linear regression on the validation data. Additionally, a pruned version of the RF models (with 50 trees instead of 200) was employed to observe the behavior of the resulting ensemble models compared to the 200-tree baseline.

Meta-learning approaches were explored beyond linear combination. A one-layer meta-learner, implemented as logistic regression, was trained to learn optimal weights for combining the base model predictions. For greater flexibility, a two-layer neural network meta-learner was also evaluated, allowing the ensemble to capture potential non-linear relationships between the base model outputs.

Finally, a gated network (GateNet) ensemble was implemented. In this approach, a small neural network was trained to dynamically assign input-dependent weights to the RF and TL predictions, effectively learning when to rely more on one model versus the other based on the input features. All ensemble models were trained and evaluated within the same LOSO cross-validation framework as the base models, ensuring fair and unbiased comparison. Pooled AUROC/AUPRC as well as two-tailed Wilcoxon tests were between ensembles and base models were calculated.

## Results

### Personalization of nociceptive signal detection

Table 1 delineates the model performances of the Random Forest and Logistic Regression baselines that are drug-naïve (physiology features only, 30 total features) and drug-aware (physiology + drug dosing features, 30 + 18 = 48 features). The RF models consistently outperform LR in terms of AUROC and AUPRC with statistical significance. Interestingly, the RF models also benefit from intraoperative drug information (AUROC 0.716 [0.700, 0.759]) versus without (AUROC 0.662 [0.640, 0.700]) with statistical significance. However, this pattern is not reflected in the LR models.

**Table 1:** Classical models versus transfer learning for nociceptive signal discrimination.

Classical	RF (aware)	RF (naïve)	LR (aware)	LR (naïve)
**AUROC** *95% CI*	0.716 [0.700, 0.759]	0.662 [0.640, 0.700]	0.623 [0.583, 0.643]	0.624 [0.602, 0.649]
**AUPRC** *95% CI*	0.399 [0.370, 0.434]	0.339 [0.309, 0.364]	0.291 [0.265, 0.317]	0.291 [0.266, 0.324]
**TL (aware)**	**1 min.**	**2 min.**	**5 min.**	**10 min.**
**AUROC** *95% CI*	0.641 [0.616, 0.663]	0.640 [0.619, 0.659]	0.639 [0.618, 0.664]	0.649 [0.619, 0.669]
**AUPRC** *95% CI*	0.317 [0.285, 0.358]	0.326 [0.301, 0.366]	0.301 [0.282, 0.365]	0.311 [0.284, 0.366]
**TL (naïve)**	**1 min.**	**2 min.**	**5 min.**	**10 min.**
**AUROC** *95% CI*	0.632 [0.611, 0.658]	0.636 [0.604, 0.651]	0.632 [0.612, 0.649]	0.638 [0.610, 0.665]
**AUPRC** *95% CI*	0.314 [0.273, 0.356]	0.316 [0.274, 0.358]	0.309 [0.272, 0.343]	0.323 [0.288, 0.346]

Model performances reported through their median AUROC/AUPRC; RF: Random Forest; LR: Logistic Regression; TL: transfer learning; aware models take 48 inputs including 18 drug features; naïve models take 30 physiologic inputs only; 95% confidence intervals reported in brackets.

**Fig 1:**
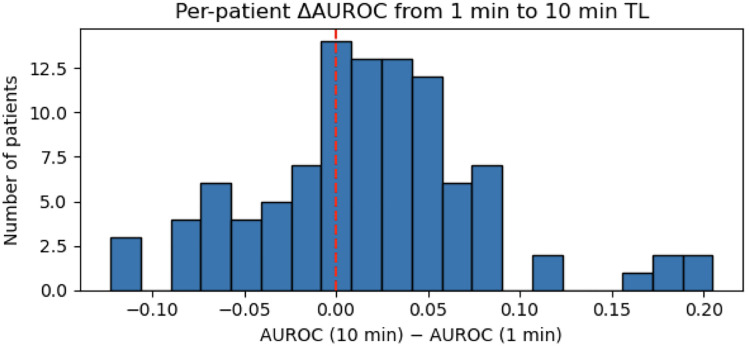
Per-patient AUROC change between 1-minute to 10-minute adaptation windows. AUROC differences were calculated for each personalized surgery (LOSO fold) then plotted in the histogram above. 66% of surgeries experienced an increase in AUROC while 34% experienced a decrease.

The performance of transfer-learning models is shown in Table 1. At face value, the models’ AUROC/AUPRCs benefit marginally without statistical significance either from drug information or increased personalization phases despite being introduced up to 10 times more data. However, a granular per-surgery AUROC benefit analysis between 10 minutes and 1 minute of personalization (Fig 1) showed that 67 of 101 surgeries (66%) improved from the additional adaptation in drug-aware models, while 34 (34%) declined. The median AUROC benefit was 0.019 with an interquartile range of [−0.023, 0.047].

### Isotonic calibration improves model accuracy

**Fig 2:**
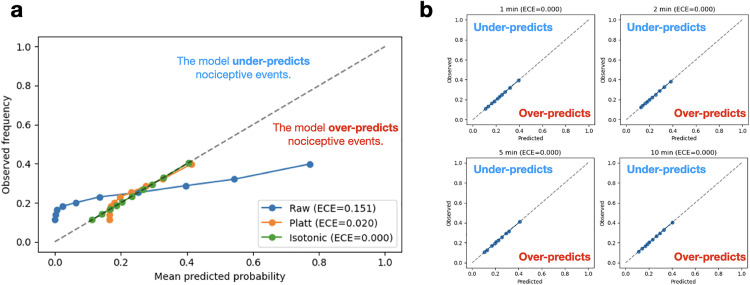
Calibration analysis of transfer-learning with varying adaptation windows. **a.** Mean-predicted probability plotted against observed frequency for the three calibration strategies. The diagonal dashed line represents the ideal calibration where observed frequency equals the predicted probability. **b.** Isotonic calibration across 1-, 2-, 5-, and 10-minute adaptation windows and their respective Expected Calibration Error (ECE).

Calibration analysis, at its simplest, is meant to show whether an **X** % predicted risk by the transfer learning model directly translates to **X** % of cases experiencing a nociceptive signal. We find that while the three calibration methods do not significantly improve its discriminatory ability in terms of AUROC (Table 2), the Brier scores and ECE indeed show significant improvement from the raw model, approaching nearly zero for all adaptation windows (Fig 2A). Platt scaling also provided some improvement (ECE_10 min_ = 0.0198), but the reliability curves still deviated from the ideal diagonal (Fig 2B), especially in the region where fewer positive samples were available. A Wilcoxon two-tailed test showed that the isotonic calibration performed significantly better than both Platt scaling and the raw models (p < 0.001).

**Table 2:** Calibration of 10-minute drug-aware transfer learning models and skipped-folds analysis.

Calibration	Uncalibrated	Platt	Isotonic
**AUROC** *95% CI*	0.649 [0.619, 0.669]	0.649 [0.632, 0.671]	0.663 [0.643, 0.671]
**Brier** *95% CI*	0.196 [0.184, 0.208]	0.158 [0.155, 0.171]	0.155 [0.151, 0.165]
**ECE** *95% CI*	0.151 [0.1318, 0.171]	0.051 [0.044, 0.056]	7.14 x 10^−9^ [5.58 x 10^-9^, 9.42 x 10^-7^]
**Adaptation Time**		**Skipped Folds (out of 101)**	
1 min.		96	
2 min.		89	
5 min.		48	
10 min.		8	

Calibration analysis of transfer-learning models and their reported median AUROC, Brier, and Expected Calibration Error scores (ECE). Statistical significance achieved in ECE scores with paired Wilcoxon in isotonic vs. Platt (p < 0.001) and isotonic vs. uncalibrated (p < 0.001). Skipped folds analysis analyze whether the adaptation windows contained sufficient information to perform Platt calibration.

Across adaptation windows, the number of “skipped folds” (Table 2)—cases where Platt scaling could not be performed due to a lack of both positive and negative events in the adaptation set—decreased substantially as the adaptation window increased. For the shortest window (1 minute), Platt scaling was feasible in only 5 out of 101 cases, with 96 folds skipped. As the adaptation window lengthened, the number of skipped folds declined, reaching 8 at the 10-minute window.

### Ensemble modeling reveals the key insights into intraoperative nociception prediction

Our baseline Random Forest models and tailored transfer-learning models were ensembled together with the hypothesis that they could not only improve performance but could also reveal key insights about the operative data and best practices when applying deep learning to nociception. The four ensemble strategies include simple linear combination, one-layer logistic regression meta-learner, two-layer non-linear meta-learner, and dynamic GateNet.

An initial comparison between a linear combination ensemble of a 200-tree RF versus a pruned 50-tree RF with the 10-minute TL model revealed that while both the RF and RF-TL ensembles outperform the TL model, there was no statistical difference in performance with increasing RF tree number (Table 3). The pruned RF(50)-TL (AUROC 0.681 [0.679, 0.684]) and unpruned RF(200)-TL (AUROC 0.683 [0.681, 0.686]) performed similarly to each other, but worse than the RF models alone. The pruned RF (AUROC 0.715 [0.713, 0.718]) and unpruned RF (AUROC 0.713 [0.711, 0.716]) also performed similarly to each other.

**Table 3:** Pooled AUROC and AUPRC comparison of RF-TL ensemble methods.

	Base models			Simple linear combination	
**Model**	**RF(50)**	**RF(200)**	**TL (10 min.)**	**RF(50)-TL**	**RF(200)-TL**
**AUROC** *95% CI*	0.715 [0.713, 0.718]	0.713 [0.711, 0.716]	0.635 [0.632, 0.637]	0.681 [0.679, 0.684]	0.683 [0.681, 0.686]
**AUPRC** *95% CI*	0.409 [0.404, 0.413]	0.415 [0.410, 0.419]	0.323 [0.319, 0.326]	0.359 [0.355, 0.363]	0.372 [0.367, 0.376]
**ENS vs. RF**				4.88 x 10^−6^	2.43 x 10^−6^
**ENS vs. TL**				8.08 x 10^–13^	2.63 x 10^–13^
	**One-layer meta-learner**		**Two-layer MLP**	**GateNet**	
**Model**	**RF(50)-TL**	**RF(200)-TL**	**RF(200)-TL**	**RF(200)-TL**	
**AUROC** *95% CI*	0.686 [0.684, 0.688]	0.708 [0.706, 0.711]	0.710 [0.708, 0.713]	0.712 [0.710, 0.715]	
**AUPRC** *95% CI*	0.365 [0.361, 0.369]	0.413 [0.409, 0.418]	0.415 [0.411, 0.420]	0.416 [0.412, 0.421]	
**ENS vs. RF**	2.53 x 10^−5^	1.70 x 10^−5^	**0.221**	**0.580**	
**ENS vs. TL**	1.26 x 10^–12^	1.09 x 10^−8^	8.69 x 10^–11^	1.65 x 10^−10^	

RF(X): Random Forest with X trees; TL: transfer-learning; RF-TL: ensemble between RF-TL; MLP: multi-layer perceptron used for ensemble; GateNet: feature-conditioned gated neural network used for ensemble; ENS vs. RF/TL: comparing ensemble performance to RF or TL using paired Wilcoxon tests on pooled AUROC. Above threshold p > 0.05 indicates no statistical difference between ensemble and base model performance.

To investigate the effects of ensemble behavior between RF and TL in nociception detection, a one-layer meta-learner was employed. Interestingly, while the pruned RF-TL (AUROC 0.686 [0.684, 0.688]) did not improve in performance, the unpruned RF-TL (AUROC 0.708 [0.706, 0.711]) performed significantly better than its pruned counterpart, despite the pruned RF(50) and unpruned RF(200) having no statistical difference. However, the unpruned RF-TL with the one-layer meta-learner still significantly underperformed than its unpruned RF counterpart (p < 0.001).

To test a further hypothesis that the interaction between model outputs may be non-linear, we utilized a two-layer meta-learner to potentially boost ensemble performance. The RF(200)-TL (AUROC 0.710 [0.708, 0.713]) performed similarly to the RF(200) (AUROC 0.713 [0.711, 0.716]) without significant difference (p = 0.221).

### Feature-conditioned gated neural networks allow ensemble interpretation

An additional ensemble strategy employing a feature-conditioned gated neural network was used to reveal data-driven permutation importance patterns of nociceptive signal prediction. The GateNet performed as well as the Random Forest (Table 3), achieving an AUROC of 0.712 [0.710, 0.715] and no statistical difference (p = 0.58). A similar per-surgery analysis revealed that 60 of 101 (59.4%) surgeries experienced an AUROC increase while 41 declined (40.6%). However, the median ΔAUROC was 0, and the IQR was [0, 0.001], suggesting spurious improvements.

**Fig 3:**
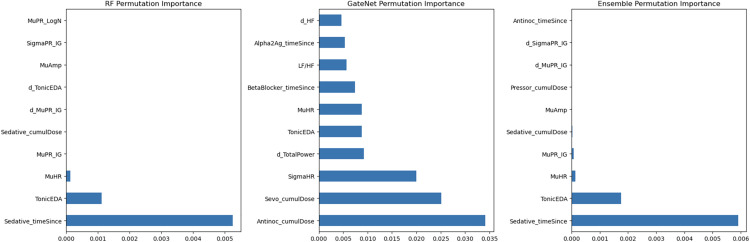
Permutation importance of predictive driver features in RF, ensemble, and GateNet. Top 10 permutation importance features for Random Forest (RF), GateNet arbitrator, and RF(200)-TL ensemble model. A higher permutation importance indicates a higher associated reliance of the model on that variable for predictive accuracy. For each feature, note that “Mu” stands for mean, “Sigma” stands for standard deviation, and “d_” indicates first derivative features.

Permutation importances were calculated from the Random Forest, RF(200)-TL ensemble, and the GateNet in Fig 3, which delineates the top 10 features from each calculation. We note that both the ensemble and Random Forest have high dependencies on time since last sedative dose, tonic electrodermal activity, and the mean heart rate, indicating a high dependency on these factors for predictive power and optimal weight assignments.

GateNet’s top three features included cumulative doses of antinociceptives, sevoflurane, and heart rate variability. Furthermore, unlike the Random Forest and the ensemble, it had proportionally similar, lesser important features such as Tonic EDA and time since beta blocker and alpha-2 agonist dosing. These features were important in determining levels of trust between the RF versus TL models.

**Fig 4:**
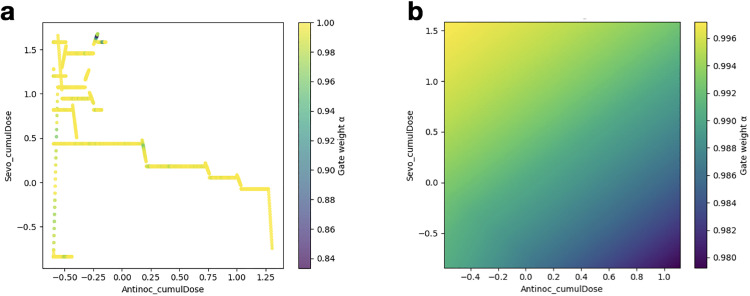
GateNet α analysis for across antinociceptive and sevoflurane dosing features. **a.** GateNet α plotted over observed evaluation windows. The colors and their corresponding α are shown in the color bar. **b.** Simulated α values across simulated evaluation windows by independently varying the respective features.

Given the high permutation importances observed with the cumulative doses of antinociceptives and sevoflurane, the GateNet α was plotted over these two features. Fig 4A depicts α over observed evaluation windows, and Fig 4B shows a heatmap of α over all simulated evaluation windows when independently varying the two features while others are held constant. Both show that α always trends near 1 with a minimum of 0.84, indicating the ensemble relied more on RF than TL to make final prediction decisions.

### Computational characterization of model complexity and scalability

To contextualize predictive performance, we performed an analytical characterization of the computational properties of the evaluated models, focusing on inference-time operations, memory footprint, and training and personalization costs.

On initial analysis, the TCN transfer-learning model was compact in terms of parameterization. The drug-aware TCN contained 20,737 trainable parameters (13,825 in the drug-naïve model), corresponding to 81 KB and 54 KB of memory under FP32 precision (four bytes per parameter), respectively. Most parameters were concentrated in the initial one-dimensional convolutional layer, with substantially fewer parameters in the subsequent normalization and dense layers.

In contrast, RF models relied on explicit tree structures rather than parametric weights. The 200-tree RF configuration was estimated to contain approximately 300,000 decision nodes, corresponding to 7.2 MB of memory, while the pruned 50-tree RF variant required 613 KB. Thus, while the TCN was substantially lighter in memory, RF models incurred a higher storage cost due to their tree-based representation.

Inference-time computational cost showed the cost of training and operationalization. For the TCN, multiply-accumulate operations (MACs) were estimated analytically from layer dimensions. The dominant cost arose from the convolutional layer, which applies 64 filters of kernel size 6 across 48 input channels, yielding approximately 18,432 MACs per inference sample after global pooling. The two fully connected layers contributed an additional (64 x 32) + (32 x 1) = 2,080 MACs, resulting in a total of 20,512 MACs per forward pass. Bias additions and activation functions were excluded from this estimate due to their comparatively negligible cost.

In contrast, RF inference involves no dense arithmetic operations; each tree performs a sequence of threshold comparisons during traversal. Given ensemble size and effective depth, RF inference required approximately 200 trees x average tree depth 10 = 2,000 logical comparisons per sample, while the pruned model required 50 x maximum tree depth 10 = 500 logical comparisons per sample. Consequently, the highest-performing RF with 50-trees with no additional personalization layers was two orders of magnitude lighter in terms of raw instruction count, favoring lower-latency and lower-power execution.

## Discussion

Accurate detection and management of pain during surgery remains a critical challenge in perioperative medicine, with significant implications for patient outcomes and the advancement of personalized care. Intraoperative nociception is inherently complex, influenced by a dynamic interplay of physiological responses and pharmacological interventions. Traditional monitoring approaches often fail to capture this complexity, leading to suboptimal pain control and increased risk of adverse events.

Prior literature has explored machine learning techniques to improve intraoperative pain assessment. However, questions surrounding the clinical utility of machine learning methodologies in the context of small data sizes and interpretability remain largely unanswered. Our study is the first to address these questions by introducing a flexible ensemble framework that integrates both classical and deep learning models, guided by a neural gating mechanism. This approach provides interpretable insights into the relative importance of physiological and drug-related features and analyzing methodologies for personalized pain detection. We harness ensemble methods and data science to assess the cost-benefit of increasing model complexity.

### Personalized deep learning doesn’t always outperform reliable supervised learning techniques

Our study found that architecture is not the only factor in creating clinically effective machine learning and artificial intelligence in precision medicine. Despite having the advantage of additional study of up to 10 minutes of a patient’s surgery in each LOSO fold, we find that transfer learning models do not outperform the Random Forest baseline. We provide several pieces of literature evidence supporting this finding.

First, the Random Forest is commonly used for performing predictions on medical tabular data [12] because of its strong performance on irregular tabular data where linear combinations of features may be uninformative for predictive power [13]. In these cases, tree-based methods like Random Forest commonly outperform deep learning methods [8,13].

Second, the models in this study were fed expertly crafted features, such as mean, standard deviations, first derivatives, drug timings, and dosages. These features may already capture high amounts of domain knowledge and pattern discovery [20]. For example, permutation importance revealed factors such as TonicEDA, mean heart rate, or standard deviation of heart rate as high importance features in our ensemble. These features have been shown to be associated with sympathetic chain function and nociceptive stimuli and response [21,22].

Against a 200-tree Random Forest trained on 100 diverse surgeries, the TCN-based transfer learning model, despite traditionally excelling on small sample sizes on prior medical detection studies [23,24], offers marginal benefits in a highly engineered feature set. In fact, fine-tuning on just the first few minutes of surgery could be detrimental, because this initial period can be noisy, not contain enough information, or not representative of the rest of the procedure. As prior studies in medical imaging have shown [25,26], if the transfer learning model overfits to this small, specific window, this may potentially make it worse at prediction compared to the global RF model.

On the other hand, our calibration analysis suggests that there may be some benefit of tuning beyond the first few minutes of surgery. This was indicated by the dramatic decrease in number of skipped LOSO folds with increasing adaptation windows, indicating increased capture of clinically relevant information.

### The unbiased judge: harnessing ensemble methods to reveal insights into medical machine learning

Ensemble methods are commonly used to mix behaviors of various models to boost the accuracy for more robust predictions [27,28]. They can also be used to reveal insights into methodologies and data.

One of the first questions we explored is the difference in performance of the ensembled RF-TL with various combination strategies and RF tree sizes. While there was no difference in performance between 50 or 200 trees, a one-layer meta-learner produced a significant difference favoring the 200-tree RF-TL. This is indicative of the increased stability (lower variance) of increasing tree size, which smooths decision boundaries at the cost of increasing computation burden and model size linearly [29].

The performance of the 200-tree RF-TL was further improved by the employment of a two-layer meta-learner. Consistent with earlier conclusions, this suggests that prediction mechanism using the engineered data is non-linear, which is suited well for RF’s robust predictive power.

This conclusion is further supported by the GateNet’s arbitrating behavior. GateNet’s top permutation importance features closely mirror that of the Random Forest model, indicating high trust. The alpha analysis suggests that in most cases, the best strategy to minimize error is to trust RF over TL, most likely due to the reasons outlined earlier in the discussion.

The clinical implications are substantial. Our findings suggests that for the purposes of nociceptive signal detection, a less computationally intensive, more interpretable, and easier-to-deploy Random Forest model is a superior approach. Especially as interpretability and scalability is at the forefront of discussion in every medical ML/AI tool, pursuing complex, “black box” models like TCNs may not be the best path forward. Instead, proper feature engineering and simple, interpretable models such as Random Forest may offer superior scalability and increase clinician trust without sacrificing accuracy.

### Computational burden

The computational characterization reinforces the central finding that increased model complexity does not necessarily translate into practical benefit in this setting. Although the transfer-learning TCN is substantially more compact in memory, its inference relies on tens of thousands of floating-point multiply-accumulate operations, whereas RF inference consists of only a few thousand simple threshold comparisons.

Combined with faster training, minimal tuning requirements, and no dependence on accelerator hardware, this makes the RF operationally cheaper and simpler to deploy despite its larger storage footprint. The TCN’s ability to rapidly adapt a small subset of parameters is theoretically appealing, but this advantage did not yield measurable performance gains in the present cohort. While GPUs can efficiently parallelize floating-point operations, the relative gap in instruction complexity remains relevant for CPU-based, embedded, or resource-constrained clinical deployment.

### Limitations

This study has limitations. The modest cohort size (101 surgeries with a ~ 6% event rate) constrains statistical power and increases susceptibility to overfitting, particularly for deep architectures. While LOSO cross-validation, early stopping, and class-weighted loss mitigate these risks, the results should be interpreted as comparative rather than definitive. A systematic ablation study was not performed due to this limitation and risk of over-partitioning the dataset and is planned for a larger follow-up study with expanded cohorts. Furthermore, permutation importance reflects model reliance rather than causal or mechanistic inference and should be interpreted as hypothesis-generating. Given the exploratory scope and limited sample size, our study focused on permutation importance as a conservative, model-agnostic baseline. Lastly, future studies should include an external validation with a secondary dataset to evaluate our findings either in methodology or in physiology.

### Final remarks

At the demonstrated accuracies, machine learning models may be clinically useful for alerting clinicians to periods of increased nociceptive likelihood or for tracking trends but is insufficient for unsupervised analgesic titration. Acceptable performance thresholds will depend on clinical context, including tolerance for false alarms, intervention costs, and integration with existing physiologic monitoring. Any deployment should therefore function within an anesthesiologist-in-the-loop framework [30], with clear visualization of contributing features to support interpretability and trust. Ethical considerations include the risk of bias driven by procedure-specific practices or pharmacologic patterns, as well as the potential for over-reliance on automated alerts.

In this comprehensive evaluation of models for nociception detection, we found that a robust, supervised Random Forest model trained on engineered physiological features established a high-performance benchmark. While deep transfer learning offers a promising paradigm for patient-specific adaptation, our results indicate it provided no significant performance gain in this setting. Furthermore, we demonstrate the utility of ensemble methods such as a gated ensemble network as a diagnostic tool, which automatically determined the marginal value of the transfer learning component to be negligible. These findings underscore the critical importance of benchmarking against strong classical models and suggest that for this clinical application, a simpler, more efficient model may be the better solution.
